# Development and Validation of a Stability-Indicating HPLC Method for the Simultaneous Determination of *trans*-Resveratrol and *cis*-Resveratrol in an Injectable Solution

**DOI:** 10.1155/2021/8402157

**Published:** 2021-11-13

**Authors:** Esmeralda Mota-Lugo, Mariana Dolores-Hernández, Elvia A. Morales-Hipólito, Iris A. Blanco-Alcántara, Hugo Cuatecontzi-Flores, Raquel López-Arellano

**Affiliations:** Laboratory of Pharmaceutical Development Tests, Multidisciplinary Research Unit, Faculty of Higher Education Cuautitlán, National Autonomous University of Mexico, Carr. Cuautitlán-Teoloyucan Km 2.5, San Sebastián Xhala, 54714 Cuautitlán Izcalli, Mexico

## Abstract

*trans*-Resveratrol, a phytochemical compound with antioxidant power and various therapeutic effects, such as cardioprotective, chemopreventive, and neuroprotective, among others, has disadvantages of poor solubility and limited stability, creating difficulties for the development of new strategies for its quantification. This study developed and validated an analytical stability method for *trans*-resveratrol by high-pressure liquid chromatography with photodiode-array detection (HPLC-PDA), which allowed its quantification in the presence of its degradation products. The quantification of *trans*-resveratrol occurred at a retention time of 2.6 min, with ammonium formate (10 mM, pH = 4)/acetonitrile, 70/30 v/v, as mobile phase. The validation met the ICH Q2 criteria of specificity, method linearity (2.8–4.2 *μ*g/ml), precision and accuracy, robustness, quantification limit (0.176 *μ*g/ml), and detection (0.058 *μ*g/ml). As degradation products, *cis*-resveratrol was observed at 3.9 min, which could be resveratrone in 3.2 min and five unidentified products in 0.7, 1.0, 1.4, 1.8, and 5 min. Some solutions subjected to temperature stress of 40 and 60°C, UV light, and acidic and basic hydrolysis exhibited colour changes. An analytical method was obtained by HPLC-PDA, which allowed quantifying the stability of *trans*-resveratrol in a fast and specific manner in the presence of its degradation products.

## 1. Introduction

**Figure 1:**
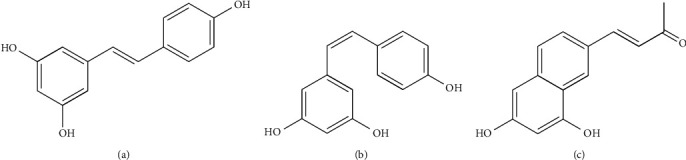
Chemical structure of compounds: *trans*-resveratrol (a), its isomer, *cis*-resveratrol (b), and its reported degradation product, resveratrone (c).

*trans*-resveratrol (3,5,4′-trihydroxystilbene; t-RSV) is a polyphenolic phytoalexin mainly found in the skin of grapes and in at least 72 species of plants, peanuts, berries, cocoa, and almonds (Figure 1(a)). It has also been found in food products such as wine, chocolate, grape juice, and peanut butter [1]. It is one of the most studied phytochemicals due to its antioxidant property [2, 3], which provides cardiovascular [4–6], chemopreventive [7–10], antiplatelet [11], neuroprotector [12, 13], and antiaging [14] effects. One of the main disadvantages of t-RSV is that it has low bioavailability [15, 16], which is a consequence of its low aqueous solubility, limited stability, and extensive liver metabolism [2, 17–19]. As a result, it represents a major obstacle for biomedical applications. The research community is currently working to solve these problems by developing new drug delivery systems that may stabilize and protect t-RSV in order to enhance its pharmacological effects [20, 21].

Some studies have indicated that t-RSV had limited stability under the influence of light, basic pH levels, and temperature, which could cause isomerization to *cis*-resveratrol (c-RSV) or t-RSV degradation. The c-RSV (Figure 1(b)) is not found naturally; it can be obtained from *trans*-resveratrol by exposure to sunlight, or by ultraviolet (UV) radiation at wavelengths of 254 nm or 365 nm [22–26]. Another degradation product reported is resveratrone (Figure 1(c)), which is a degradation product of the isomer, c-RSV, that only occurs in ethanol solutions and acidic conditions, but not in water [24, 26, 27].

Most analytical methods reported for the identification and quantification of t-RSV have been applied in wine [28–30] or in pharmacokinetic studies [19, 31–33]. These methods are based on gas chromatography [29, 34], with tandem mass spectrometry detection and fluorometry [35, 36]. However, some disadvantages of these methods are their high equipment costs and long retention times [23, 25, 28, 30, 37, 38]. In contrast, studies that used high-pressure liquid chromatography-UV-visible (HPLC-UV/Vis) to deal with formulation development have only addressed analytical methods development, but not full validation. Until now, there are very few studies that addressed the quantification of t-RSV in the face of its degradation products generated by forced degradation [25, 39], and most of them have focused on photolytic studies with UV light [22–24, 26, 40].

Stability is the most important quality requirement for a pharmaceutical product. It allows a better choice of the components that will make part of the formulation and the appropriate packaging. Stability will also provide optimal storage conditions and expiration dates [41]. This way, the identity, effectiveness, potency, safety, and purity of pharmaceutical products will be assured until the moment of use. Quantification methods used in stability studies should be specific; i.e., they should be capable of quantifying only the substance of interest, without interference from some other component or degradation product of the sample. The International Conference on Harmonisation (ICH) provided recommendations in the guideline Q1A (R2) for conducting degradation and stress tests [42]. Liquid chromatography has been widely used to develop methods for determining stability. It has advantages over other methods with respect to the separation of all compounds in a single run. In addition, this method promotes easy identification of impurities and degradation products [43].

Considering the low stability and solubility of t-RSV, the hypothesis of the present study was that selection of the optimal conditions of the analytical method and its validation would allow the identification and specific quantification of *trans*-resveratrol in the face of different conditions of forced degradation. The goal was to develop an analytical method using high-pressure liquid chromatography (HPLC) coupled to a photodiode-array detector (PDA) that allowed the quantification of *trans*-resveratrol in a parenteral formulation in the presence of its isomer (c-RSV) and its degradation products. The method created was validated with respect to linearity, selectivity, precision and accuracy, robustness, and quantification and detection limit. *trans*-RSV was assessed under acidic, basic forced degradation, oxidative degradation, and photo degradation, as well as the effects of different temperatures.

## 2. Materials and Methods

### 2.1. Materials

The following chemicals and reagents were used in the present study: resveratrol (99% purity), Sigma-Aldrich® (St. Louis, MO, USA); resveratrol, raw material (50% purity) Alephquim® (CDMX, MX); PEG 400 and PG; ammonium formate (AF) (99.0% purity), Sigma-Aldrich® (St. Louis, MO, USA); RA-grade formic acid (89.2% purity), Tecsiquim® (CDMX, MX); HPLC-grade acetonitrile (ACN) (99.9% purity); ACS-grade methanol (MetOH) (99.9% purity); ACS-grade ethanol (EtOH) (99.9% purity); ISO-grade hydrogen peroxide (H_2_O_2_) (31% purity), Fermont® (Mty, MX); sodium hydroxide (NaOH) (98.4% purity); RA-grade hydrochloric acid (HCl) (37.8% purity); and RA-grade glacial acetic acid (HAc) (99.9% purity), JT Baker® (MA, USA). The water used in all the experiments was filtered using the Milli-Q water purification system (18.2 MΩ), Millipore equipment.

### 2.2. Equipment

The samples were assessed using the following equipment: Waters® HPLC system; Waters® 616 quaternary pump; multisolvent delivery controller system Waters® 6CE 600 controller; Waters® 717 plus autosampler; and Waters® 996 photodiode-array detector. Data collection and processing were performed using the Empower 2.0 software.

Robustness of the method was assessed using Waters® ACQUITY UPLC H-class system, equipped with Waters® quaternary multisolvent delivery controller system, Waters® FNT autosampler, CHA column heater, and Waters® PDA e*λ* UPL diode array detector. Data collection and processing were performed using Empower 3.0 software, version 7.1, 2010.

For the photo-stability test, the samples were placed in the UVLS-24 Model UV Lamp (UVP) chamber; under the effect of temperature at 40°C, the samples were placed in a MAPSA oven, HDP-433 model and at 60°C in a Felisa oven, model 133.

### 2.3. Optimization Chromatographic Conditions

Halo® C18 columns (3.0 × 75 mm, 2.7 *μ*m, USA) were used at a flow of 0.3 ml/min and an injection volume of 10 *μ*l, containing 21 *μ*g/ml of t-RSV raw material for the selection of the mobile phase. Different mixtures of MetOH, ACN, HAc (pH = 4; 0.25%), and AF (10 mM) acidified with formic acid (pH = 4) were used to choose the mobile phase. Once the mobile phase was chosen, the t-RSV response was compared with different columns: Agilent® Zorbax Rapid Resolution High Definition (RRHD) SB-C18 (4.6 × 50 mm, 1.8 *μ*m, USA); Agilent® Zorbax Eclipse XDB C8 (4.6 × 50 mm, 1.8 *μ*m, USA); Waters® Symmetry C18 (4.6 × 75 mm, 3.5 *μ*m, Ireland); Inertsil® ODS-3 C18 (33 × 46 mm, 3 *μ*m, India); and Phenomenex® Luna C18 (150 × 4.6 mm, 5 *μ*m, USA), conditioning the flow for each column with a pressure of 1200 psi.

### 2.4. Chromatographic Conditions

The Waters® Symmetry C18 column (4.6 × 75 mm, 3.5 *μ*m, Ireland) was used with 10 mM ammonium formate mobile phase, acidified at pH = 4 with formic acid and acetonitrile, 70/30 v/v. The mobile phase had a flow of 0.9 ml/min and a run time of six minutes. The aliquot of the injected sample was 10 *μ*l. The chromatograms were obtained at a wavelength of 307 nm, since it is the maximum absorbance of t-RSV. The percentage of t-RSV concentration (% t-RSV) was calculated using (1), where *A*_std_ corresponds to the peak area of the standard and *A*_sample_ to the peak area of the sample:

(1) $$\begin{array}{c} \%\text{ t}-\text{RSV}=\frac{A_{\text{std}}}{A_{\text{sample}}}\times 100. \end{array}$$

### 2.5. Preparation of Standards and Sample Solution

#### 2.5.1. Standard Solution

The initial standard solution of t-RSV had a concentration of 21 *μ*g/ml, which was diluted with a mixture of EtOH/water, 1/1 v/v, to prepare six standard solutions in a range of 0.525–16.8 *μ*g/ml for the calibration curve.

#### 2.5.2. Parenteral Solutions

Formulation 1 (F1) and Formulation 2 (F2) contained 3.5 mg/mL of t-RSV, both formulations developed in the laboratory. The placebo of F1 consisted of a mixture of EtOH/water/PEG 400, 10/30/60 v/v/v, whereas the placebo of F2 consisted of a mixture of EtOH/water/PG, 15/25/60 v/v/v.

#### 2.5.3. Sample Solutions Added

Five placebos were prepared with resveratrol, in a final concentration range of 2.8–4.2 *μ*g/ml, corresponding to 80–120% of t-RSV. Each solution was prepared independently and randomly.

#### 2.5.4. Sample for Forced Degradation

Standard t-RSV (STD), F1, F2, and their corresponding placebos were used for the forced degradation method. They were diluted to a final concentration of 4.2 *μ*g/ml of t-RSV. For the effect of temperature, photolysis was diluted with EtOH/water, 1/1 v/v. Acid hydrolysis was diluted with 0.1 N HCl, basic hydrolysis with 0.1 N NaOH, and oxidation with 3% H_2_O_2_. Each solution was prepared independently and randomly.

### 2.6. Validation of the Method

The validation of the analytical method was performed in accordance with the recommendations established in the ICH Q2 guideline [44].

#### 2.6.1. Specificity

Samples in triplicate of diluent solution were used as blank. Samples of solutions corresponding to 100% concentration of t-RSV, standard solution, placebo added of F1 and F2, placebo of both solutions, and samples of F1 and F2 were subjected to UV light stress (*λ* = 365 nm), 0.1 N NaOH, 0.1 N HCl, 3% H_2_O_2_, and a temperature of 60°C for 24 hours (h). In order to determine the specificity, it was first confirmed that there was no analytical response in the analyte retention time.

#### 2.6.2. System Linearity

A 6-level calibration curve was performed in triplicate and independent of t-RSV standard solutions (0.525–16.8 *μ*g/ml). Linearity was determined by linear regression analysis, calculating the correlation coefficient, determination coefficient, relative standard deviation, and confidence interval of the *y*-intercept.

#### 2.6.3. Precision and Accuracy

The results and discussion may be presented separately, or in one combined section, and may optionally be divided into headed subsections.

#### 2.6.4. Linearity of the Method

Linearity was assessed using a calibration curve in triplicate, with five concentration levels, in a range of 80–120% of placebos with t-RSV, which were prepared independently and by individual weighing. The recovery percentage and the relative standard deviation were determined, and a linear regression analysis was performed calculating the correlation coefficient, determination coefficient, the confidence intervals of the *y*-intercept, and the slope in order to determine the linearity of the method.

#### 2.6.5. Quantification Limit and Detection Limit

The limit of detection (LOD) and limit of quantification (LOQ) were determined using the standard deviation of the *y*-intercept of the regression (*S*_*β*_0__) and the value of the slope (*β*_1_), withboth values of the calibration curve, using

(2) $$\begin{array}{c} \text{LOD}=3.3\times \left(\frac{S_{\beta_{0}}}{\beta_{1}}\right), \end{array}$$

(3) $$\begin{array}{c} \text{LOQ}=10\times \left(\frac{S_{\beta_{0}}}{\beta_{1}}\right). \end{array}$$

#### 2.6.6. Robustness

The effect of intentional variations in the analytical conditions was assessed, namely: injection volume (10 ± 1 ml); mobile phase AF 10 mM pH = 4 and ACN (70/30 ± 1%); pH of the mobile phase of AF 10 mM (pH = 4 ± 1); and equipment change to UPLC. For the assessment, six loaded placebos corresponding to 100% of the analyte were analysed for each condition, calculating the absolute difference of the arithmetic mean corresponding to the area of the normal condition and to the modified condition.

### 2.7. Stress Degradation Studies

The forced degradation study was conducted with samples of STD, F1, F2, and placebos, which were diluted to a 4.2 *μ*g/ml concentration. The forced degradation effect was obtained under acidic, basic, and oxidative conditions, at temperatures ranging from 40 to 60°C, and the photolysis effect (UV light, *λ* = 365 nm) and temperature effect (4, 25, 40, and 60°C) were assessed for 120 h, in accordance with the recommendations established in the ICH Q1A and Q1B guidelines [42, 45]. The analyte was quantified from 0, 24, 72, and 120 h of exposure to the degradation conditions.

#### 2.7.1. Kinetic Study

For the kinetic analysis, the t-RSV concentration was measured at different time intervals (0, 24, 72, and 120 h). The recovery percentages of t-RSV were used to determine the degradation kinetics for each sample, using first order equations and logarithm of the percentage of recovered t-RSV concentration (log% RSV) as a function of time in h (*T*), respectively [46], represented by (4), where *K*_0_ represents the value of the unadjusted degradation constant, a value that was used to get the adjusted degradation constant (*K*_*A*_) obtained from the Arrhenius graph, given by *P*, which are percentiles taken at two or more temperatures of the study, following an Arrhenius model (5), where *T* is the temperature in Kelvin degrees, *B* = 1/11605 (Boltzmann's constant), and *A* and *E* are two unknown parameters [47].

(4) $$\begin{array}{c} \log\;\%\text{RSV}=-K_{0}\times T, \end{array}$$

(5) $$\begin{array}{c} K_{A}=P=A^{\left(-E/\left(B\times T\right)\right)}. \end{array}$$

The procedure estimates the Arrhenius model and extrapolates a percentile for a normal operating temperature [47], in which the estimated percentile represents *K*_*A*_. The time of 90% concentration (t90) was estimated using (6), where *C*_0_ represents the initial concentration of t-RSV and *K*_*A*_ represents the estimated degradation constant.

(6) $$\begin{array}{c} \text{t}90=\frac{\log\left(C_{0}/0.9\right)\times C_{0}}{K_{A}}. \end{array}$$

### 2.8. Data Analysis

All experiments were performed in at least triplicate, and data are expressed as mean ± relative standard deviation (% RSD). The results were statistically assessed by analysis of variance with 5% significance level (*p* < 0.05), using the statistical software STATGRAPHICS Centurion version XV.II (https://Statgraphics.Net, Madrid, Spain).

## 3. Results and Discussion

### 3.1. Optimization Chromatographic Conditions

Retention times (*t*_*R*_), capacity factor (*k*′), width (*w*), and peak area (*A*) were compared for the development of the method and its optimization. First, four mobile phases were tested with the Halo C18 column, namely: MetOH/HAc (pH = 4; 0.25%), 60/40 v/v; ACN/HAc (pH = 4; 0.25%), 30/70 v/v; ACN/Water/MetOH, 30/35/35 v/v/v; and CAN/FA acidified with formic acid, pH = 4, 10 mM, 60/40 v/v, choosing the last mentioned mobile phase, due to better chromatographic parameters (*t*_*R*_ = 1.6 min, *w* = 81 s, *A* = 873672, *k*′ = 2.35) in comparison to those of the other mobile phases. Then, once the mobile phase was chosen, the column was selected assessing the Halo C18, Zorbax C18, Zorbax C8, Symmetry C18, ODS-3 C18, and Luna C18 columns. Two flow rates were used: one of 0.2 ml/min in the Zorbax C18, ODS-3, and Zorbax C8 columns and the other of 0.8 ml/min in the Luna C18, Halo C18, and Symmetry C18 columns, both columns with a pressure of 1200 psi (Figure S1 in supplementary information). The Symmetry C18 column was chosen for exhibiting the best chromatographic results in comparison to those of the other columns. It featured the shortest retention time (*t*_*R*_ = 2.6 min), a symmetric well-defined peak, a narrow peak (*w* = 21 s, *A* = 2715729), and a *k*′ of 4.27.

**Figure 2:**
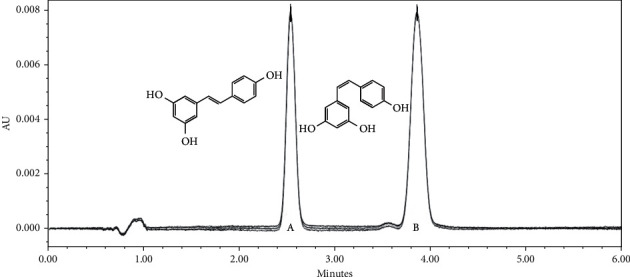
Chromatogram of *trans-/cis-*resveratrol. The following were obtained from the final working conditions: 10 mM acetonitrile/ammonium formate mobile phase (pH = 4), 30/70 v/v, at a flow of 0.9 ml/min; t-RSV at a *t*_*R*_ of 2.6 min (peak “*A*”); and c-RSV at a *t*_*R*_ of 3.9 min (peak “*B*”), with Symmetry C18 column.

Subsequently, adjustments of the flow rate and the phase proportion were made using the Symmetry C18 column, mobile phase of ACN/FA (pH = 4, 10 mM) 60/40 v/v, and a sample of t-RSV subjected to UV light (*λ* = 254 nm) for seven days to generate c-RSV. The purpose was to obtain the best resolution of the peaks and the shortest running time. The conditions with the best resolution were 70% of 10 mM ammonium formate acidified with formic acid at pH = 4 and 30% acetonitrile at a flow of 0.9 ml/min, with a resolution value of 2.91 and a *t*_*R*_ of 2.6 min for t-RSV and 3.9 min for c-RSV (Figure 2).

### 3.2. Validation of the Method

The method optimized for the assessment of t-RSV stability was validated according to the established guidelines of the ICH Q2. The results obtained are shown in Table 1, and they met the acceptance criteria of the said guidelines.

**Figure 3:**
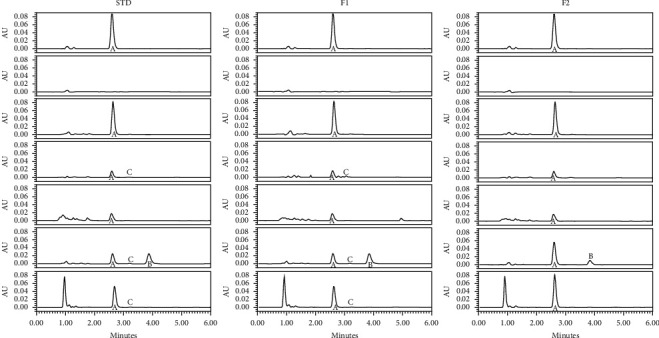
Specificity test of the method. Chromatograms of samples of t-RSV STD, F1, and F2 are observed without stress (I), their placebos (II), and under conditions of forced degradation for 24 h at a temperature of 60°C (III), acid hydrolysis (IV), basic hydrolysis (V), UV light (VI), and oxidation (VII). The peak “A” is *trans*-resveratrol, peak “B” is its isomer, and *cis*-resveratrol and peak C are believed to be resveratrone.

The analysis of the samples subjected to stress for 24 h to assess the specificity of the method indicated the presence of degradation products. However, no additional signals were observed in the *t*_*R*_ and analyte signal. This way, it was determined that there was no interference in the quantification and identification of t-RSV and that the signal was only due to the analyte (Figure 3). The linearity of the system obtained a correlation coefficient of 0.999 and a coefficient of determination of 0.998, with % RSD of 1.39, criteria considered acceptable. The confidence interval of the *y*-intercept included zero. Subsequently, accuracy and precision obtained a recovery percentage of 100.16 ± 1.47% and a percentage of residues less than 2, as indicated in the guidelines. The linearity of the method obtained a recovery percentage of 100.11 ± 1.20% and RSD of 1.19%. The confidence interval of the *y*-intercept did not include zero, whereas the confidence interval of the slope included unity. Regarding LOD and LOQ, the values obtained were 0.058 and 0.176 *μ*g/ml of t-RSV, respectively. The robustness was demonstrated by changes in the proportion of the mobile phase, change in pH of the mobile phase of AF 10 mM, and change of equipment, in comparison to normal conditions, expressed with its recovery percentage and its absolute value of arithmetic difference.

### 3.3. Stress Degradation Studies

The chromatograms of the method indicative of stability confirm the efficiency of the methodology developed and validated by HPLC coupled to DAD used to assess the stability of resveratrol in parenteral formulation (Figures S2–S6 in supplementary information). It can be observed that the detection was specific of t-RSV during the stability study performed, in which the peak of the analyte decreased due to forced degradation, but did not exhibit signal interference by any type of degradation product in the *t*_*R*_ of the analyte, in any of the assessed conditions. Therefore, the created method was specific and suitable for the intended use. Furthermore, the placebos subjected to stability did not indicate the presence of degradation products or signals interfering with the t-RSV response.

**Figure 4:**
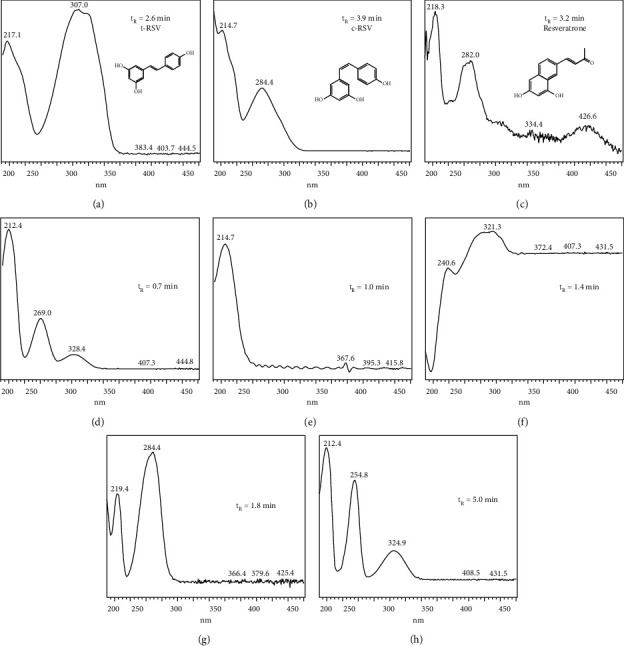
Spectra of degradation products of t-RSV. The spectrum of t-RSV (a) at a retention time (*t*_*R*_) of 2.6 min, its isomer at 3.9 min, c-RSV (b), and its degradation product at 3.2 min, which is believed to be resveratrone (c).

Figure 4 illustrates the absorption spectra obtained from the chromatographic peaks of the t-RSV degradation products. It is worth mentioning that t-RSV had its *t*_*R*_ = 2.6 min, and the maximum absorption was 307 nm (Figure 4(a)). The first peak, a degradation product of t-RSV, was at *t*_*R*_ = 0.7 min, exhibiting maximum absorbance at 212, 269, and 328 nm (Figure 4(d)). It was found in the chromatograms from the 24 h under basic hydrolysis conditions in F1 and F2, under UV light in F1, on 72 h at temperatures of 40 and 60°C in F1, and in the STD sample at 4 and 25°C on 120 h under UV light. At minute 1.0, there was another degradation peak, with maximum absorbance at 214 nm (Figure 4(e)). It was observed from the 24 h in the chromatograms of the effect of the temperature in F1 at 40°C, in all acid and basic hydrolysis samples, and in UV light in F1 and F2, whereas in the STD, it was observed until the 120 h of analysis. The peak at *t*_*R*_ = 1.4 min exhibited maximum absorbance at 240 and 321 nm (Figure 4(f)), and it was observed in the chromatograms from 24 h in all samples of basic conditions, acid hydrolysis at 40°C, and UV light of F1, from 72 h under acidic hydrolysis conditions at 40°C in F2, on 120 h in the t-RSV standard sample subjected to UV light, and at 60°C in F1 and F2. At *t*_*R*_ = 1.8 min, another peak was observed with maximum absorbance of 219 and 284 nm (Figure 4(g)). It was observed in the chromatograms of the effect of temperature at 60°C on 72 h in F2 and on 120 h in F1, in acid hydrolysis at 40°C in F1 from 24 h and in F2 on 72 h, whereas, in basic hydrolysis, it was observed in all samples from 24 h. At the *t*_*R*_ of 3.2 min, the degradation peak exhibited maximum absorbance of 218, 282, and 426 nm (Figure 4(c)), which was believed to be the degradation product of t-RSV, identified as resveratrone [26]. However, further analyses should be performed with mass spectrometry or magnetic resonance imaging to elucidate the molecule. This peak was observed in the chromatograms of the effect of temperature at 60°C in F1 and F2 on 120 h, in acid hydrolysis at 40°C in F2, and under UV light from 24 h; however, it was not very significant in these chromatograms. The peak corresponding to c-RSV was observed at a *t*_*R*_ of 3.9 minutes, with a maximum absorbance of 284 nm (Figure 4(b)), from 24 h in all samples exposed to UV light. The last peak was observed at a *t*_*R*_ of 5.0 min (Figure 4(h)), with maximum absorbance of 212, 264, and 324 nm, observed in the standard sample on 24 h in ambient light, and on 120 h in the formulation 1 in dark environment.

Table 2 illustrates the values obtained from the recovery percentages of t-RSV after 120 h, the adjusted degradation rate constants (*K*_*A*_), and the life time at 90% of t-RSV (t90), which were obtained from the first order graph of the percentage logarithm of recovered concentration of t- RSV (log% t-RSV) with respect to time, and the Arrhenius graph at each degradation condition, respectively.

**Figure 5:**
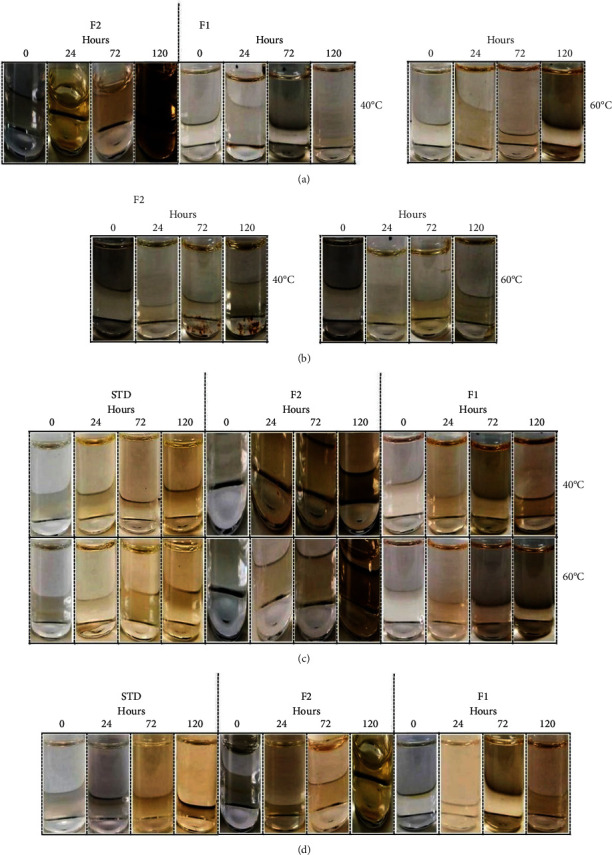
Colour change. Study on sample in t-RSV STD, F2, and F1 solutions subjected to forced degradation in temperature (III), acid hydrolysis (IV), basic hydrolysis (V), and exposure to photolysis with UV light at *λ* = 365 nm (VI). Note: the solutions that did not exhibit colour changes were not included in the image.

On the other hand, it is worth mentioning that colour change was observed in some solutions subjected to stress (Figure 5). There was a colour trend; when the active principle (i.e., t-RSV) began to degrade, the colourless solution turned into yellow-orange, which darkened as the days went by, until it reached a brown-gray colour. In the literature, there are no data on colour changes in t-RSV solutions caused by degradation; however, it can be attributed to the nature and composition from which the raw material of the active principle comes, the excipients, and the interaction caused by their degradation products in the solution.

Regarding the results under acid hydrolysis conditions, F2 had greater degradation at a temperature of 40°C, reaching 36.58% recovery on 24 h, in comparison to the results obtained at 60°C, with 52.53% recovery after 120 h. On the other hand, F1 exhibited greater stability in comparison to F2, a fact that may have occurred due to the excipients, with a recovery percentage close to 12% at both temperatures on the 120 h. However, the standard obtained lower degradation percentage than the formulations, recovering 88.06% at 40°C and 81.06% at 60°C on 120 h. The standard obtained a t90 of 174.91 h and 77.86 h at 40 and 60°C, respectively. F2 had a t90 of 20.2 h at 60°C, and 2.51 h at 40°C. In the case of F1, a t90 of 7.02 and 5.85 h were estimated for 40 and 60°C, respectively. The F2 solutions were those that exhibited colour change (Figure 5-IV), which turned yellow at both temperatures (40 and 60°C), during the 120 h. On the other hand, in addition to yellow, the solutions at 40°C exhibited red/brown precipitate on 72 and 120 h. This formulation rapidly degraded at a temperature of 40°C, exhibiting 0% recovery of t-RSV on 72 h ―when the precipitate appeared―which could denote that the solid corresponded to a degradation product of the analyte.

With respect to basic hydrolysis, it was observed that, at 40°C, F2 had better stability, with a recovery percentage of 41.39%, whereas STD and F1 tended to degrade rapidly, with 4.41% recovery on 24 h, and 19.44% on 72 h, respectively. At a temperature of 60°C, it was observed that the three samples featured the same stability pattern, obtaining on 24 h a recovery percentage of 5.49% of STD, 1.39% for F1, and 0.0% in F2. At the temperature of 40°C, there were t90 values of 2.46, 2.89, and 1.54 hours for STD, F2, and F1, respectively, whereas, for the temperature of 60°C, the t90 values obtained were 0.88, 0.22, and 0.58 h. According to the values obtained, it was considered that, in the three samples assessed, basic hydrolysis exhibited a higher rate (%) of degradation in comparison to that of acid hydrolysis. It can be seen that t-RSV STD had better stability under acidic conditions, whereas, under basic conditions, F2 was more resistant to degradation. Figure 5-V shows that the standard exhibited, both at 40 and 60°C, a yellow coloration during the 120 h of assessment. F1, at 40 and 60°C, exhibited a yellow coloration on 24 h, whereas on 72 and 120 h it had a translucent brown-gray coloration. From its preparation, the solutions in formulation 16 turned translucent yellow (it was not possible to capture the colour in the photograph). On 72 and 120, at a temperature of 40°C, the solutions were translucent yellow-orange and, at 60°C, they turned translucent brown.

Oxidation with 3% H_2_O_2_ indicated that, at 40°C, on 120 h, F1 exhibited better stability, with a recovery percentage of 43.59%, followed by F2 with 29.42%, whereas the standard degraded faster, with 15.43% recovery. The recoveries obtained at a temperature of 60°C were 28.12% on 24 h for STD, 18.35% for F1, and 0.01% for F2. The t90 value for STD, at 40°C, was 7.06 h, and at 60°C 2.05 h. For F2 40°C, it was 9.93 h and at 60°C 0.27 h. In the case of F1, t90 were 16.54 h and 1.49 h for 40 and 60°C, respectively. In this case, the formulations and the standard suffered a very rapid degradation; however, the F1 sample, at 40°C, exhibited slower oxidation in comparison to F2 and STD, which had no colour changes.

With respect to temperature effect, it was observed that t-RSV exhibited better stability at 4°C, temperature at which higher recovery percentages were obtained on 120 h, which were greater than 95% in the three samples. At 25°C on 120 h, the STD and F2 samples showed the same degradation trend, recovering more than 90%, whereas for F1 the degradation rate was faster, with 88.76% recovery. At 40°C, the degradation was greater for F2, recovering 65.23% on 120 h, whereas F1 exhibited the lowest degradation value (75.84%). At 60°C the degradation on 120 h for the standard was slower (67.76% recovery) in comparison to the samples of F1 and F2, which achieved 4.40 and 8.31% recovery, respectively. At 4°C, the STD sample exhibited the highest t90, with 540.24 h, whereas F1 had the lowest (457.94 h). Regarding ambient temperature (25°C), the standard sample exhibited the highest value, with a t90 of 146.77 h. The lowest value was observed in F1, with a t90 of 79.68 h. At 40°C, the t90 of the standard was 64.39, whereas those of formulations 16 and 2 were 29.60 and 26.38 h, respectively. At 60°C, the t90 of F2 and F1 were similar, i.e., 7 h, whereas the t90 of the standard was 24.09 h. In the case of thermal conditions of 60°C (Figure 5-III), it was observed in F1 that, on 24 h, the solution exhibited a yellowish colour, on 72 h, it turned orange, and, on 120 h, it turned dark exhibiting a greyish-brown coloration. At 40°C, from 24 to 120 h, F2 exhibited a translucent yellow coloration (it was not possible to capture the colour correctly in the photograph), at 60°C, from 24 to 72 h, it showed a translucent orange coloration and on 120 h translucent brown coloration.

In the photolysis test, on 120 h, in the absence of light (i.e., darkness), the F2 and F1 samples had similar degradation behaviour (84.47% and 85.02%), whereas the STD obtained recovery of 97.48%. In ambient light, STD and F1 exhibited similar degradation values. Both formulations recovered about 85% on 120 h, whereas for F2 the recovery was 36.51%. In UV light, at a wavelength of 365 nm, on 120 h, F2 was the one that obtained the lowest degradation percentage, with 30.35% recovery, whereas the STD and F1 samples showed very similar degradation kinetics, recovering 12.26 and 11.19%, respectively. In darkness, t90 values of 701.17, 87.93, and 71.76 h were obtained for STD, F1, and F2, respectively. In ambient light, the t90 for STD was 90.51 h, in F1 85.35 h, and in F2 13.24 h. In UV light, t90 of 7.43, 6.98, and 11.64 h were obtained for STD, F1, and F2, respectively. The solutions that exhibited colour changes were those exposed to UV light at *λ* = 365 nm (Figure 5-VI). On 24 h, F1 and F2 exhibited a light orange coloration, on 72 h it was yellow, and on 120 h the colour was light brown. The standard solutions exhibited a yellow coloration on 72 and 120 h.

## 4. Conclusions

An analytical method indicative of stability HPLC was developed. It allowed quantifying t-RSV in parenteral formulation and raw material. It turned out to be fast, simple, and specific in the presence of its degradation products. In addition, it featured shorter times for retention, running, limit detection, and quantification in comparison to methods reported in the literature. The method assessed in the present study met the validation parameters established in the ICH Q2 validation guidelines. Its efficiency and selectivity were confirmed by the indicative stability method, which revealed that the stability of t-RSV samples was changed by basic hydrolysis, temperatures greater than 40°C, and UV light (*λ* = 365 nm). It was also possible to detect seven degradation products, from which their absorption spectrum was obtained. Colour change from translucent to yellow-orange was detected in the solutions subjected to forced degradation. The constant degradation for each t-RSV sample and its concentration time were determined by means of the first order equation. The samples were more stable at a temperature of 4°C and in the absence of light.

## Figures and Tables

**Table 1:** Summary of validation parameters of t-RSV.

Parameter	Acceptance requirements						
	*r* (>0.998)	*r* ^2^ (>0.995)	% recovery^A^ (98–102%)	% RSD (<2.0)	IC (*β*_0_) includes 0	IC (*β*_1_) includes 1	|*d*_*i*_| (<2)
Linearity of the system^B^	0.999	0.998	^*∗*^	1.39	−4729.09 at 1448.42	^*∗*^	^*∗*^
Accuracy and precision^C^	^*∗*^	^*∗*^	100.16 ± 1.47	1.47	−0.001 at 0.25	0.93 at 1.00	^*∗*^
Linearity of the method^C^	0.999	0.998	100.11 ± 1.20	1.19	−0.06 at 0.23	0.94 at 1.02	^*∗*^
Robustness							
Mobile phase composition (FA/ACN)	69/31 v/v		100.36 ± 0.51	0.51	^*∗*^	^*∗*^	0.32
	71/29 v/v		99.89 ± 0.38	0.39	^*∗*^	^*∗*^	0.16
pH of FA	pH = 3		100.00 ± 0.81	0.81	^*∗*^	^*∗*^	0.00
	pH = 5		100.00 ± 0.35	0.35	^*∗*^	^*∗*^	0.00
Changing equipment	UPLC		100.01 ± 0.24	0.24	^*∗*^	^*∗*^	0.01

*r* = correlation coefficient; *r*^2^ = determination coefficient; IC (*β*_0_) = *y*-intercept confidence interval; % RSD = relative standard deviation; IC (*β*_1_) = slope confidence interval; |*d*_*i*_| = absolute difference. ^A^Mean % recovery ± standard deviation; ^B^ = standards; ^C^ = fortified samples. ^*∗*^Acceptance requirements are not required for the validation parameter.

**Table 2:** Results of the forced degradation study of t-RSV samples: STD, F1, and F2.

Condition		*T* (°C)	STD				F1				F2			
			*K*_*A*_ (×10^−3^ h^−1^)	*t*_90_ (h)	*r* ^2^	% recovery (120 h)	*K*_*A*_ (×10^−3^ h^−1^)	*t*_90_ (h)	*r* ^2^	% recovery (120 h)	*K*_*A*_ (×10^−3^ h^−1^)	*t*_90_ (h)	*r* ^2^	% recovery (120 h)
Acid hydrolysis, 0.1 N HCl		40	0.60	174.91	0.91	88.06 ± 0.42	14.95	7.02	0.56	12.89 ± 0.46	41.87	2.51	0.75	36.58 ± 0.64^a^
		60	1.35	77.89	0.99	81.06 ± 0.23	17.95	5.85	0.98	12.03 ± 0.64	5.20	20.20	0.91	52.53 ± 0.14

Basic hydrolysis, 0.1 N NaOH		40	42.75	2.46	0.74	4.41 ± 1.839^b^	68.17	1.54	0.62	19.44 ± 1.40^a^	36.30	2.90	0.76	41.39 ± 1.04^a^
		60	119.55	0.88	0.50	5.49 ± 1.21^a^	181.05	0.58	0.47	1.30 ± 1.47^a^	479.50	0.22	0.46	0.00 ± 0.43^a^

Oxidation, 3% H_2_O_2_		40	14.88	7.06	0.99	15.43 ± 1.15	6.35	16.54	0.86	43.52 ± 0.28	10.57	9.93	0.96	29.42 ± 0.70
		60	51.29	2.05	0.69	28.12 ± 0.82^a^	70.57	1.49	0.61	18.35 ± 0.61^a^	383.88	0.27	0.46	0.01 ± 0.34^a^

Temperature		4	0.19	540.24	0.99	98.06 ± 0.53	0.23	457.94	0.86	95.37 ± 0.66	0.16	650.77	0.94	97.23 ± 0.76
		25	0.72	146.77	0.89	91.27 ± 0.46	1.32	79.68	0.98	88.76 ± 0.42	1.07	97.99	0.96	93.53 ± 0.32
		40	1.63	64.40	0.95	70.79 ± 1.92	3.98	26.38	0.98	75.84 ± 0.40	3.55	29.60	0.97	65.23 ± 0.91
		60	4.36	24.09	0.97	67.76 ± 0.69	14.89	7.05	0.99	4.40 ± 0.84	14.80	7.10	0.81	8.31 ± 0.50

Photolysis	Darkness	25	0.15	701.17	0.90	97.48 ± 0.60	1.19	87.93	0.86	85.02 ± 0.60	1.46	71.76	0.96	84.47 ± 0.98
	Environment		1.16	90.51	0.86	85.60 ± 0.98	1.23	85.35	0.95	84.95 ± 1.21	7.93	13.24	0.82	36.51 ± 0.73
	UV light *λ* = 365 nm		14.11	7.43	0.51	12.26 ± 1.07	1.50	6.98	0.51	11.19 ± 0.40	9.02	11.64	0.84	30.35 ± 0.44

*K*_*A*_ = adjusted degradation constant; *t*_90_ = 10% RSV degradation time; *r*^2^ = determination coefficient; % recovery = mean ± standard deviation (*n* = 3). ^a^t-RSV concentration recovery after 24 h; ^b^t-RSV concentration recovery after 72 h.

## Data Availability

The data used to support the findings of this study are included within the article.

## Supplementary Materials

Supplementary Materials
*Figure S1*. Chromatograms of t-RSV in the evaluated columns. *Figure S2*. Chromatograms of the effect of acid hydrolysis. *Figure S3*. Chromatograms of the effect of basic hydrolysis. *Figure S4*. Chromatograms of the oxidation effect. *Figure S5.* Chromatograms of the effect of light. *Figure S6*. Chromatograms of the effect of temperature.
