# Supplementary material for: Development and Validation of a Stability-Indicating HPLC Method for the Simultaneous Determination of trans-Resveratrol and cis-Resveratrol in an Injectable Solution
Source: J Anal Methods Chem. 2021 Nov 13;2021:8402157. doi: 10.1155/2021/8402157 (PMC8605923; doi:10.1155/2021/8402157)
Supplement: Supplementary Materials — Figure S1. Chromatograms of t-RSV in the evaluated columns. Figure S2. Chromatograms of the effect of acid hydrolysis. Figure S3. Chromatograms of the effect of basic hydrolysis. Figure S4. Chromatograms of the oxidation effect. Figure S5. Chromatograms of the effect of light. Figure S6. Chromatograms of the effect of temperature. [file 8402157.f1.pdf]

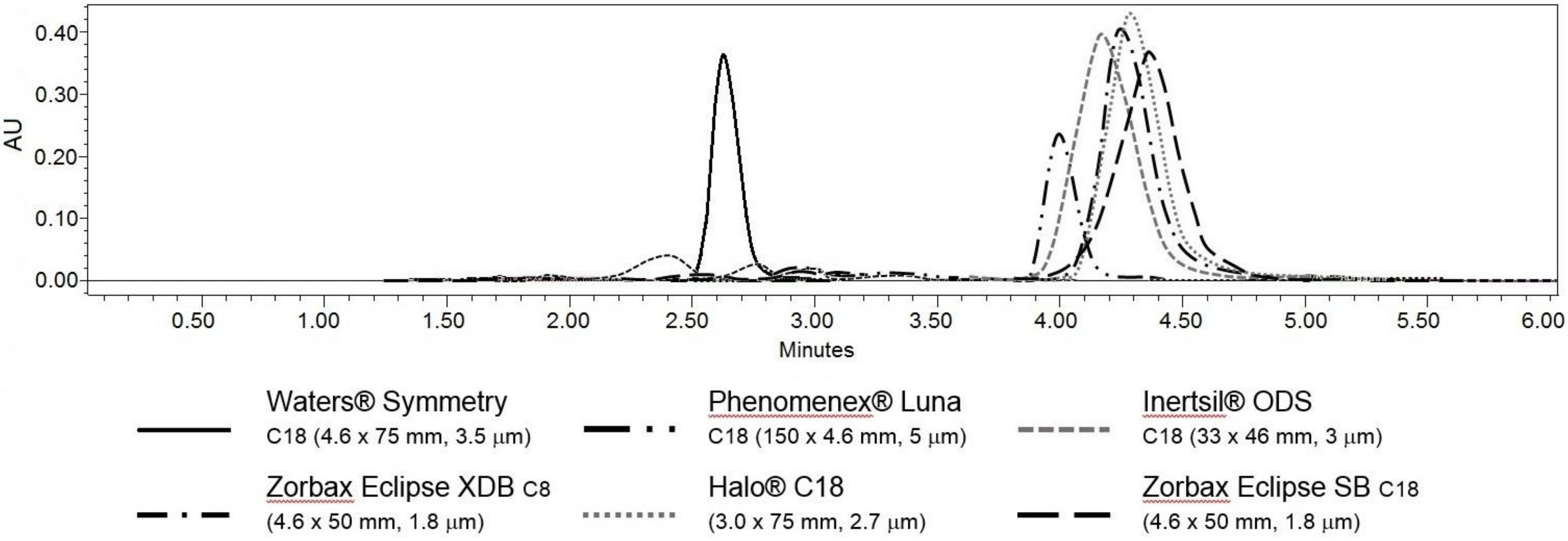

Figure S1. Chromatograms of t-RSV in the evaluated columns.

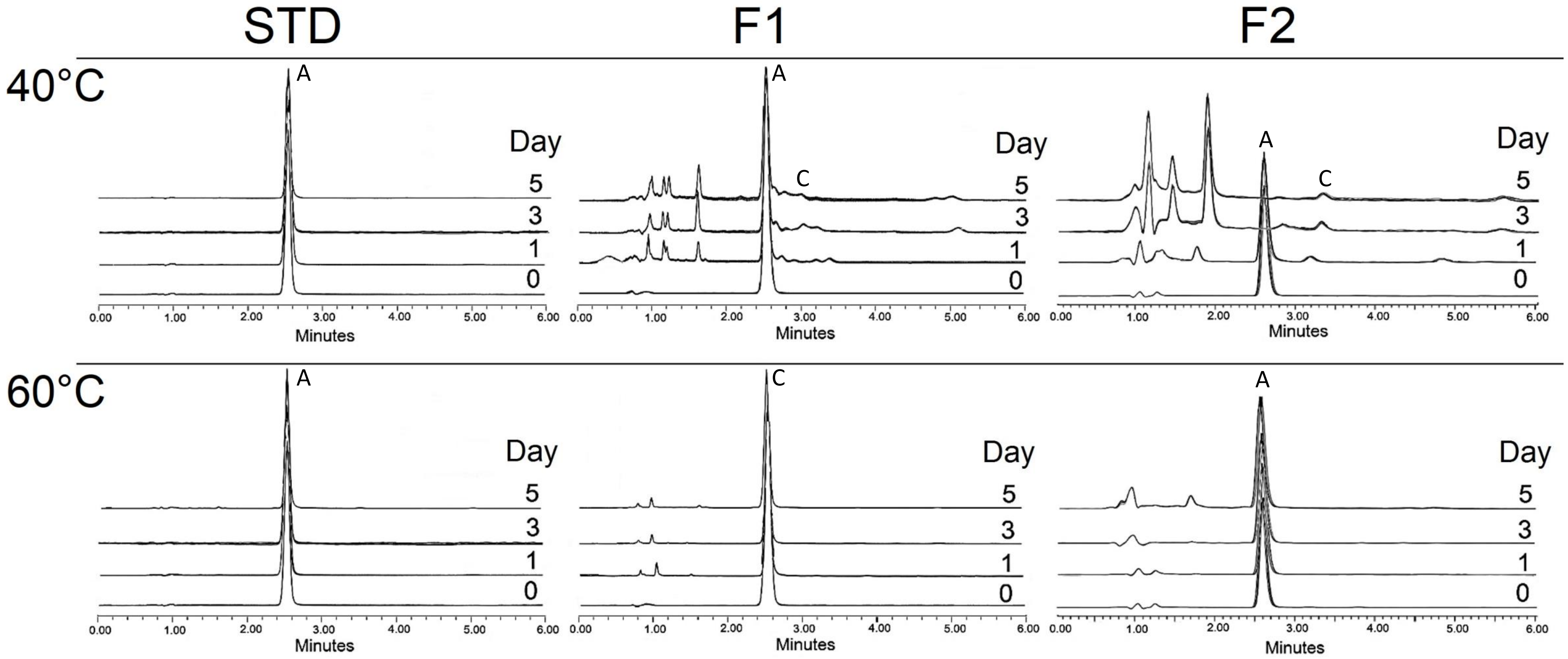

Figure S2. Chromatograms of the effect of acid hydrolysis.Study on samples of t-RSV STD, F1, and F2 during the 5 days exposure to forced degradation, with 0.1 N HCl at 40 °C and 60 °C. The peak “A” is *trans*-resveratrol and peak “C” which was believed as resveratrone.

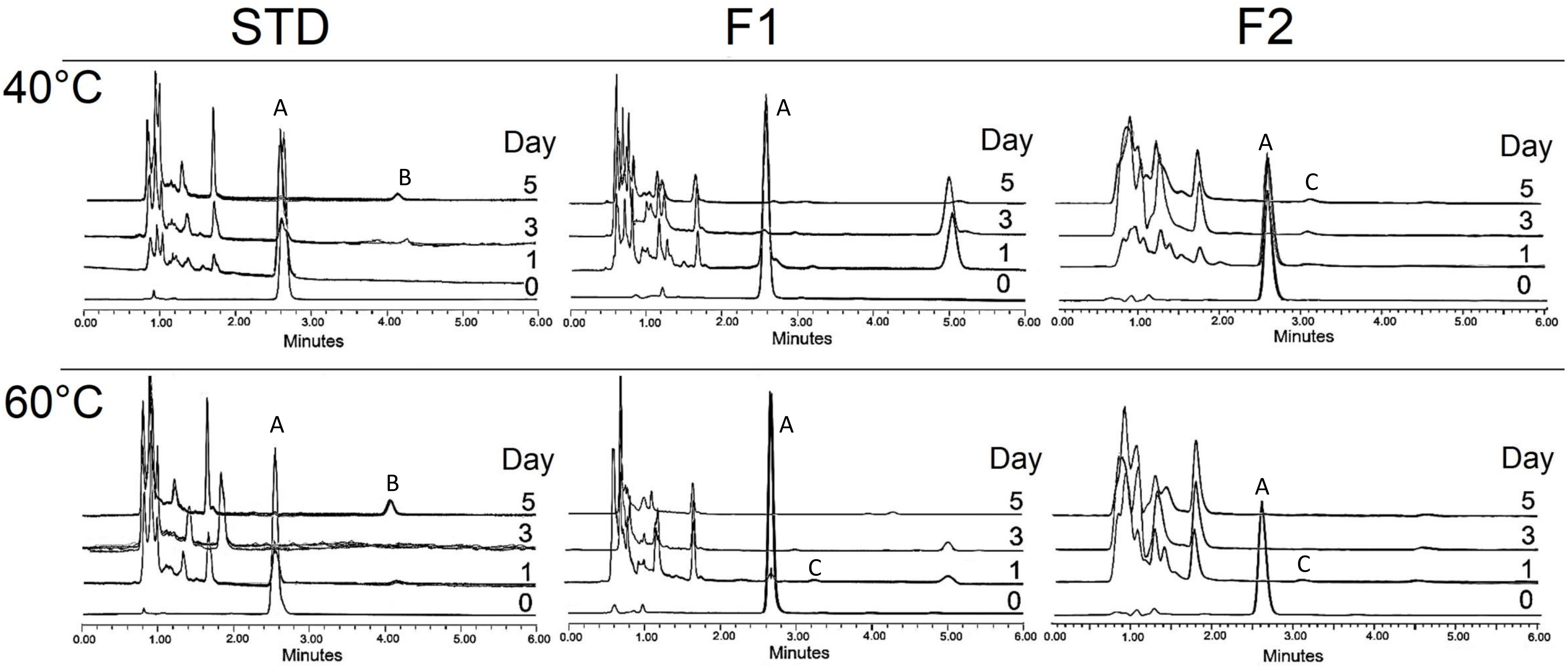

Figure S3. Chromatograms of the effect of basic hydrolysis. Study on samples of t-RSV STD, F1, and F2 during the 5 days exposure to forced degradation, with 0.1 N NaOH at 40 °C and 60 °C. The peak “A” is *trans*-resveratrol, peak “B” its isomer, cis-resveratrol and peak “C” which was believed as resveratrone.

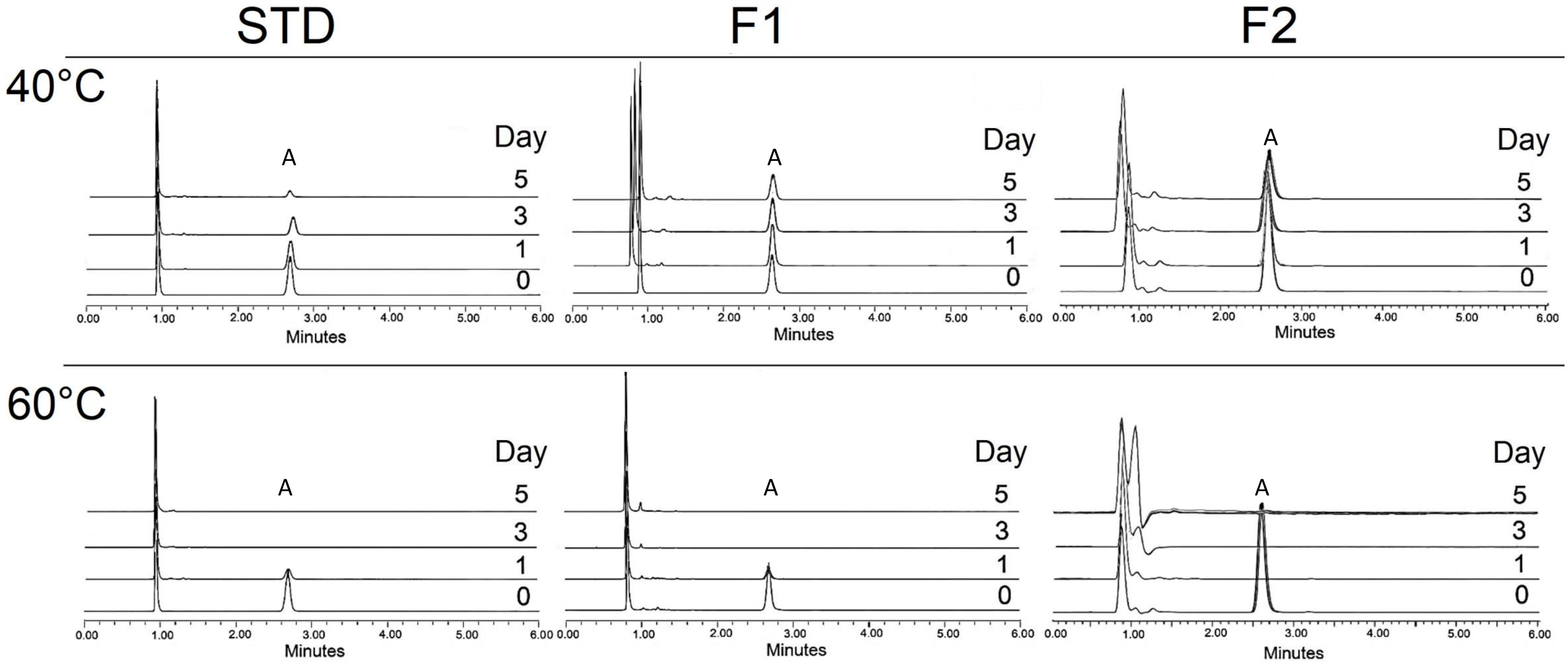

Figure S4. Chromatograms of the oxidation effect. Study on t-RSV STD, F1, and F2 samples during the 5 days exposure to forced degradation, with 3% H_2O_2 at 40 °C and 60 °C. The peak “A” is *trans*-resveratrol.

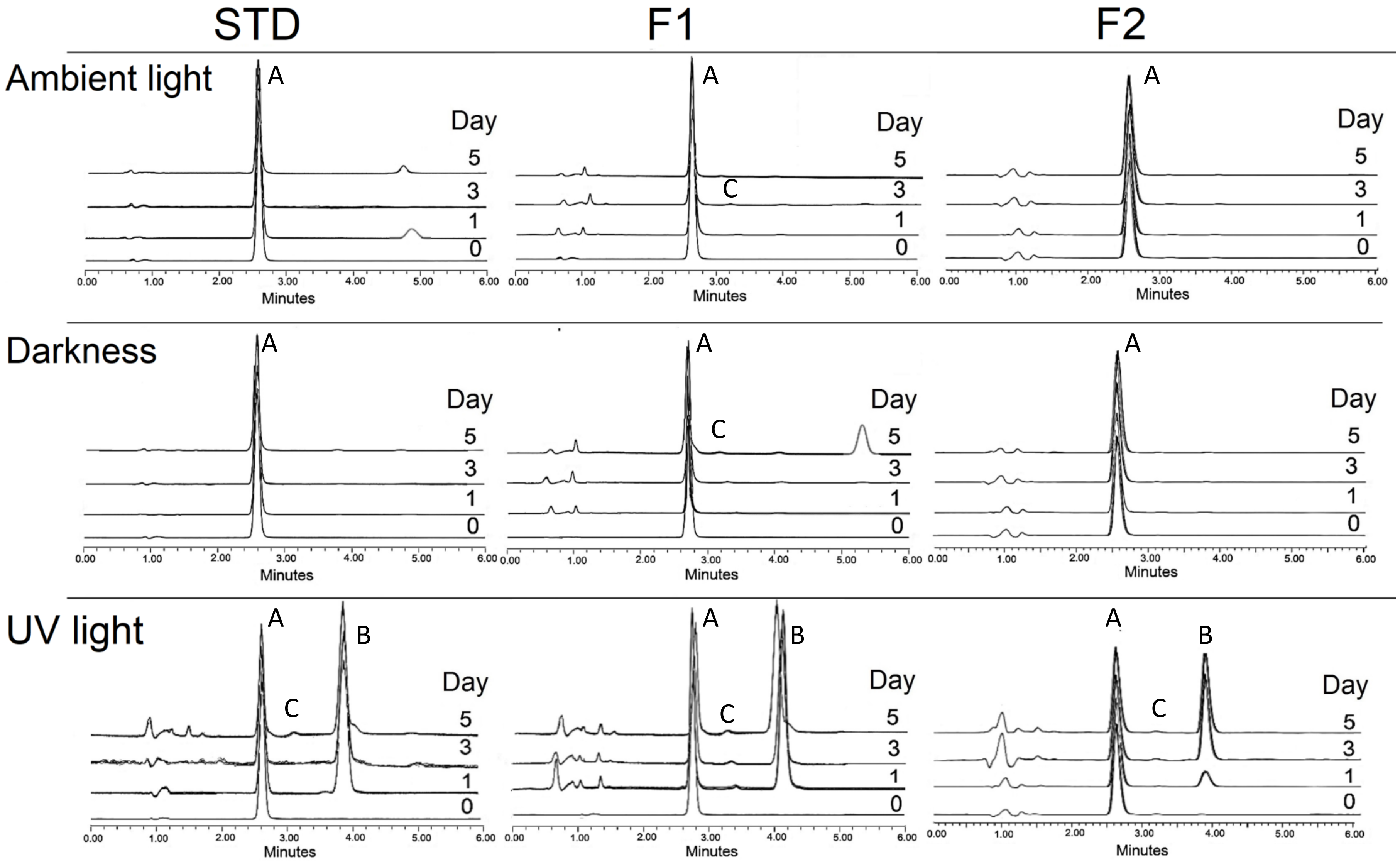

Figure S5. Chromatograms of the effect of light. Study on samples of t-RSV STD, F1, and F2 during the 5 days exposure to forced degradation under ambient light, darkness and UV light. The peak "A" is *trans*-resveratrol, peak "B" its isomer, cis-resveratrol and peak "C" which was believed as resveratrone.

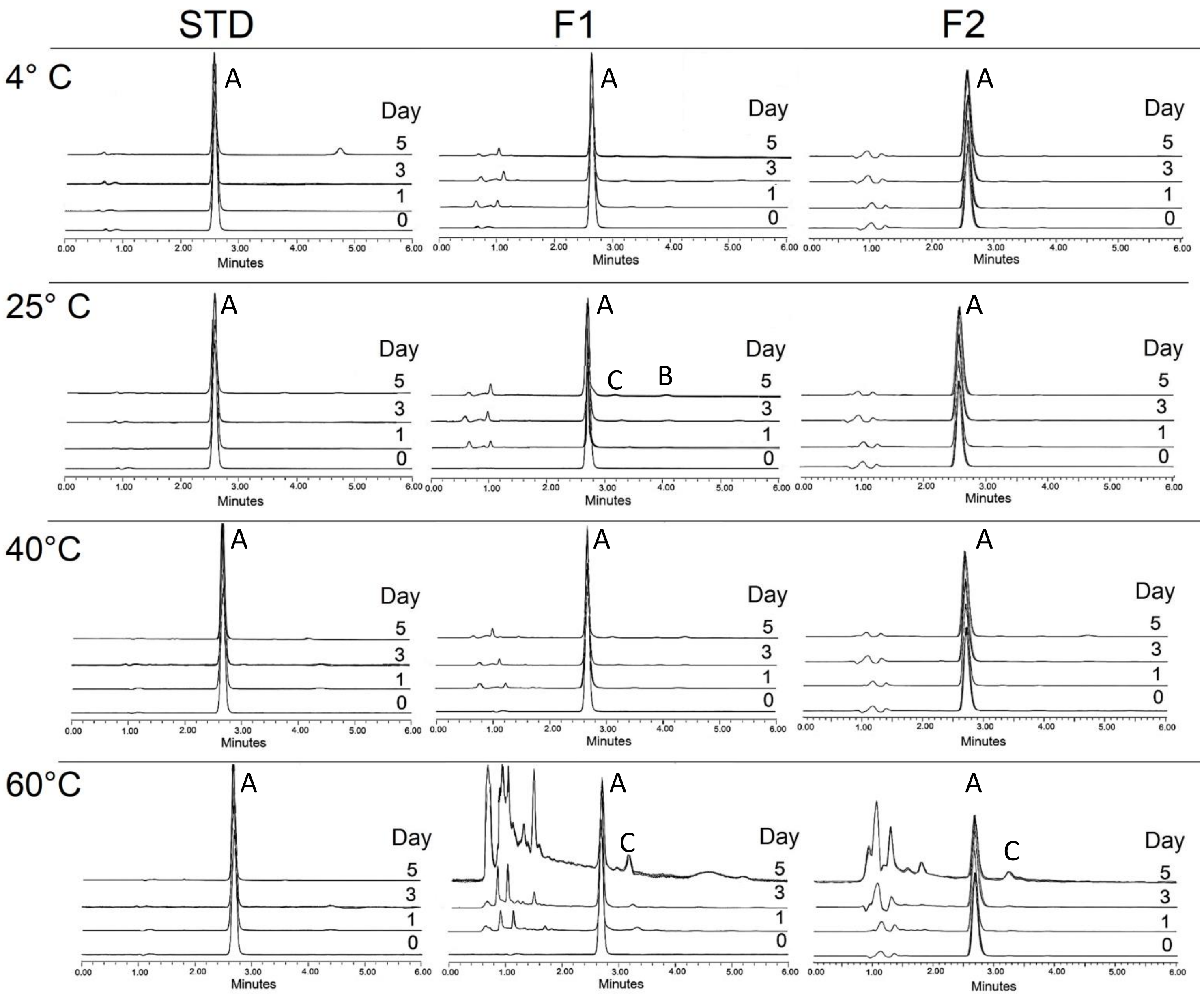

Figure S6. Chromatograms of the effect of temperature. Study on samples of t-RSV STD, F1, and F2 during the 5 days exposure to forced degradation at 4, 25, 40, and 60 °C. The peak "A" is trans-resveratrol, peak "B" its isomer, cis-resveratrol and peak "C" which was believed as resveratrone.
